# PB.20. Audit of 5 years of routine screening mammography of the reconstructed breast

**DOI:** 10.1186/bcr3724

**Published:** 2014-11-03

**Authors:** A Ray, L McNeilly, A Newland, S Flais

**Affiliations:** 1 Ealing Hospital, Southall, UK

## Introduction

In 2008, allowing for an increase in the number of women treated with mastectomy and immediate reconstruction, the decision at our institution was made to carry out mammography of the native and the reconstructed breast at follow-up. A literature review at that time revealed that mammography of the reconstructed breast may help diagnose recurrence before any symptom. This local practice is now audited to evaluate its impact, and whether it should continue.

## Methods

All women who had previously undergone mastectomy and reconstruction, and were subsequently imaged with mammography between 2008 and 2013 at Ealing Hospital were included. Subsequent findings and further investigations have been analysed.

## Results

Eighty patients treated with mastectomy and reconstruction had regular screening mammograms. Ten local recurrences occurred in six patients. Four recurrences were diagnosed on imaging findings alone (three on mammography, one on MRI). Six recurrences presented with a new symptom (palpable lump or skin abnormality). Routine imaging of the reconstruction generated additional imaging: seven stereobiopsies for microcalcifications (two recurrences) and two ultrasound scans (one recurrence). In the same period, 17 ultrasound scans (five recurrences) and 12 MRIs (one recurrence, but not consistent with the symptom) were requested for clinical concerns.

## Conclusion

Routine imaging of the reconstructed breast can diagnose local recurrence with limited harm, generating few additional investigations. However, which patients may benefit and whether this improves outcome is unclear as the number of cases in our audit is small and the event is thankfully rare.

